# Langerhans cell histiocytosis misdiagnosed as liver cancer and pituitary tumor in an adult: A case report and brief review of the literature

**DOI:** 10.3892/ol.2014.1928

**Published:** 2014-02-28

**Authors:** JING MA, YONGFANG JIANG, XIANGYU CHEN, GUOZHONG GONG

**Affiliations:** Department of Hepatitis Diseases, Second Xiangya Hospital, Central South University, Changsha, Hunan 410011, P.R. China

**Keywords:** Langerhans cell histiocytosis, Langerhans cell, liver, spleen, pituitary

## Abstract

Langerhans cell histiocytosis (LCH) is a rare proliferative disorder in which pathological Langerhans cells accumulate in a variety of organs. LCH usually affects the bone, skin and lymph nodes of children; however, LCH occasionally affects vital organs, including the liver, spleen and pituitary gland. The present study reports a case of an adult LCH patient with marked liver damage, splenomegaly and pituitary damage treated using a new therapeutic strategy. This case was misdiagnosed as liver cancer and pituitary tumor on the basis of abdominal ultrasound, abdominal magnetic resonance imaging (MRI) and head MRI. The final diagnosis was established by identifying the proliferation of cluster of differentiation 1a-positive LCs in liver tissues. A new regimen of combined 12-week therapy of prednisolone/desmopressin/vincristine and 10 months of maintenance therapy of prednisolone/vinblastine/6-mercaptopurine improved symptoms, liver function and blood cell tests.

## Introduction

Langerhans cell histiocytosis (LCH) is a rare disease of unknown etiology characterized by mixed cellular infiltration with colonial proliferation of LCs in specific histopathological lesions, which results in a variety of clinical manifestations. LCs are identical to normal dendritic cells. These atypical and immature cells of the mononuclear phagocytic system can infiltrate virtually anywhere in the body and may occur in localized lesions or as widespread systemic diseases (1). LCH has an extremely variable presentation that depends on abnormal proliferation and dissemination of histiocytes. Therefore, LCH has numerous clinical forms that affect different systems or different sites in the same system with variable outcomes (2). LCH occurs in the bones, skin and mucous membranes in children, and occasionally in other organs in adults.

Depending on the extent and localization of the disease at the time of evaluation, LCH can be classified as single system LCH when one organ/system is involved or multisystem LCH (MS-LCH) when two or more organs/systems are involved. MS-LCH is reported in <30% of LCH cases (3). The hematopoietic system, liver and/or spleen are considered high-risk organs rich in histiocytes. LCH has been reported in two to five cases per million individuals annually and rarely occurs in adults with liver or pituitary lesions (1). This study reports an adult LCH patient with liver and pituitary dysfunction as well as spleen damage. To the best of our knowledge, manifestation of LCH in the liver, spleen and pituitary gland of an adult has not been reported previously.

Diagnosis of LCH is based on histological and immunophenotypic examination of lesional tissue. The key step is the morphological identification of the characteristic LCs. Positive cluster of differentiation (CD)1a and/or Langerin (CD207) staining in lesional cells is required for definitive diagnosis (4). LCH has variable clinical symptoms, as it affects a wide variety of systems (2). Specific symptoms include pain, swelling, skin rashes, otorrhea, irritability, fever, loss of appetite, diarrhea, polydipsia, dyspnea and behavioral and neurological changes (5). Currently, there is no standard treatment for LCH. Age, extent of disease and dysfunction of vital organs are major factors that need to be considered in deciding treatment. Due to the fact that no previous standard of care exists for the treatment of LCH, the Histiocyte Society developed the Histiocyte Society Evaluation and Treatment Guidelines in April, 2009 (6). According to the guidelines, LCH patients with multiorgan involvement and dysfunction of the liver, lungs or bone marrow are considered a high-risk group. An initial six-week course of therapy with vinblastine and prednisone is recommended for all patients with multisystem disease. Further therapy depends on patient response to the initial therapy. Written informed consent was obtained from the patient.

## Case report

A 45-year-old male visited the Second Xiangya Hospital (Changsha, China) with complaints of fever, fatigue, anorexia, jaundice and polyuria for two months. Physical examination revealed that body temperature and heart rate were 38.3°C and 95 bpm, respectively, with moderately stained skin and sclera. The spleen was found to be enlarged 3 cm below the costal margin. The patient exhibited slight pain in the region of the liver. The patient had no bone pain or superficial lymphadenopathy. No abnormalities were detected on heart and lung examination.

Laboratory testing revealed significantly elevated levels of alanine transaminase (200 U/l; normal range, 0–40 U/l), aspartate transaminase (256 U/l; normal range, 0–37 U/l), total bilirubin (110 *μ*mol/l; normal range, 5.1–17.1 *μ*mol/l), direct bilirubin (89 *μ*mol/l; normal range, 0–6.0 *μ*mol/l), alkaline phosphatase (1,005 U/l; normal range, 30–110 U/l), γ-glutamyltranspeptidase (2,547 U/l; normal range, 11–50 U/l) and lowered urinary specific gravity (1.0; normal range, 1.005–1.030). The patient was negative for hepatitis viruses A, B, C and E.

**Figure 1:**
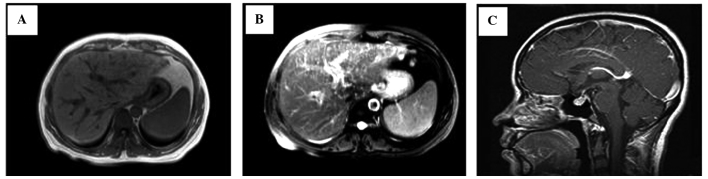
Contrast-enhanced magnetic resonance imaging (MRI) (A and B) T1 signal of the liver. (A) Prior to the injection of the contrast agent and (B) following the injection of a contrast agent. (C) T2 signal of the head.

Abdominal ultrasound revealed enlarged spots on the liver and splenomegaly. Abdominal MRI revealed splenomegaly and the typical characteristics of diffuse liver cancer. For example, the contrast-enhanced T1 signal revealed an uneven signal from the liver parenchyma, miliary diffuse nodules and no expansion of the intrahepatic bile duct (Fig. 1A). Enhanced early-phase scanning indicated uneven perfusion, arterial-phase scanning revealed significantly enhanced nodules, and venous- and delayed-phase scans were marginally enhanced, indicating the typical changes of liver cancer (Fig. 1B). Head MRI revealed an abnormal skull shape with a thickened and enlarged pituitary stalk >3 mm (Fig. 1C). The patient was diagnosed with liver cancer and pituitary tumor.

**Figure 2:**
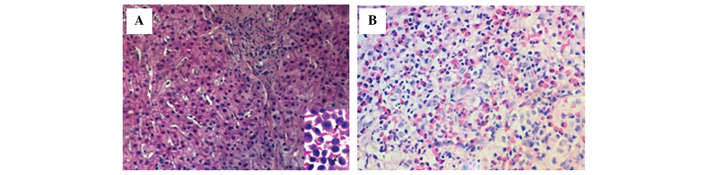
Histological staining. (A) Hematoxylin and eosin-stained liver section; (B) positive immunohistochemical staining of cluster of differentiation 1a in Langerhans cells (red). Magnification, ×40.

However, liver histopathology revealed proliferation of LCs, infiltration of eosinophils and other inflammatory cells, including a number of foam cells and multinucleated giant cells (Fig. 2A). Immunohistochemical staining revealed CD1a-positive LCs (Fig. 2B). Therefore, the patient was diagnosed with LCH, liver and spleen lesions, as well as central diabetes insipidus.

The patient was treated with prednisolone (40 mg/m^2^/day), desmopressin (0.1 mg/m^2^/day) and vincristine (6 mg/m^2^/week) for six weeks. During this period, the symptoms of fever, fatigue and anorexia improved. Volume of urine was reduced to 2,000–3,000 ml from an initial 6,000–8,000 ml every 24 h, but liver function tests revealed no significant improvement. The initial therapy phase was extended for another six weeks. During the following six weeks, oral prednisolone (40 mg/m^2^/day) was administered three days per week with vincristine (pushed 2 mg/m^2^/week). The patient was followed-up for 10 months with treatment consisting of prednisolone (40 mg/m^2^/day, days 1–5, Q3 weeks), vinblastine (6 mg/m^2^/day, Q3 weeks) and 6-mercaptopurine (MP) (50 mg/m^2^/day for seven months). As a result of the treatment, liver function and blood cell tests improved.

## Discussion

In the present case, liver damage was diagnosed by skin and scleral jaundice as well as abnormal liver function tests. Abdominal ultrasound and MRI revealed the typical changes of liver cancer. The final diagnosis of LCH for this case mainly depended on histological identification of LCH cells and CD1a-positive staining of LCs. In addition, this particular case had three characteristics that have rarely been reported previously: i) LCH occurred in an adult with damage to three vital organs (the liver, spleen and pituitary gland); ii) damage to the bone (which occurs in 92% of cases) and skin (24% of cases), two of the most commonly affected organs, was not detected; iii) a regimen of 12-week therapy of prednisolone/desmopressin/vincristine plus maintenance therapy of prednisolone/vinblastine/6-MP resulted in marked improvement in symptoms and liver function (7).

LCH is a group of idiopathic disorders characterized by the presence of cells with features similar to bone marrow-derived LCs accompanied by a backdrop of hematopoietic cells, including T-cells, macrophages and eosinophils. LCH occurs in all age groups but usually affects children between one and 15 years old, with peak incidence between five and 10 years of age. Liver images reveal nodular and irregular lesions, and spleen images demonstrate splenomegaly (enlargement >2 cm below the costal margin in the midclavicular line) (8). Radiological manifestations of the disease include thickening of the pituitary stalk >3 mm, loss of physiological hyperintense signal in the posterior pituitary on T1W images, and loss of antidiuretic hormone storage granules (9). Diagnosis is confirmed histologically by tissue biopsy. Appropriate histopathological tests are required to establish a diagnosis of LCH. Heterogeneous admixture of cells is also revealed by microscopy, including eosinophils, polymorphonuclear leukocytes, giant cells and mononuclear cells, as well as fibrosis. In cases of LCH, numerous mononuclear cells are LCs, distributed diffusely or in clusters, which supports the diagnosis (10). The immunohistochemical confirmation of the presence of LCs by cell surface CD1a antigen is useful for diagnosis. The CD1a surface antigen can now be identified from routinely paraffin-embedded specimens (11).

Thus far, the precise pathogenesis has not been elucidated and there is no standard treatment for LCH. Currently, there are four basic types of therapy for patients, including debridement surgery, radiation therapy, chemotherapy and immunotherapy, depending on the affected vital organs. However, radiation therapy is only suitable for limited lesions, not for multisystem disease. Due to the lack of a standard of care for the treatment of LCH, three large-scale, international, prospective therapeutic studies for multisystem LCH were conducted by the Histiocyte Society in April, 2009 (6). LCH is divided into high- and low-risk groups with different treatments for each. The low-risk group is defined as patients older than two years without liver, bone marrow or lung involvement. Patients with multiorgan involvement and dysfunction of the liver, lungs or bone marrow, and lack of response to initial therapy (assessed after 6–12 weeks of treatment) are placed in the high-risk group. Therefore, the patient reported in the present study fits the characteristics of the high-risk group.

Different regimens for the treatment of adult LCH with multisystem involvement have been reported with variable efficacy (12). Natural mortality of LCH is high, although patients have improved prognosis when presenting with bone and skin lesions. Patients also have improved prognosis should they respond to chemotherapy during the first six weeks of treatment, regardless of whether there is multiple organ damage. For the present case, a 12-week regimen of prednisolone/desmopressin/vincristine was followed by maintenance therapy of prednisolone/vinblastine/6-MP. Although this regimen resulted in marked improvement in symptoms and liver function, the patient did not respond well to the initial six-week therapy, and no noticeable improvement in liver function was detected at first. After 10 months of maintenance therapy, however, results of liver function tests revealed significant improvement. To the best of our knowledge, this represents a new treatment regimen. Therefore, this study reports a rare case with multifocal disease and a unique treatment. Although the patient was treated with a unique therapy and survived for more than one year, further study is required.

## References

[1] Leonidas JC, Guelfguat M, Valderrama E (2003). Langerhans’ cell histiocytosis. Lancet.

[2] Gasent Blesa JM, Alberola Candel V, Solano Vercet C (2008). Langerhans cell histiocytosis. Clin Transl Oncol.

[3] García Gallo MS, Martínez MP, Abalovich MS (2010). Endocrine manifestations of Langerhans cell histiocytosis diagnosed in adults. Pituitary.

[4] Camelo-Piragua S, Zambrano E, Pantanowitz L (2010). Langerhans cell histiocytosis. Ear Nose Throat J.

[5] Howarth DM, Gilchrist GS, Mullan BP, Wiseman GA, Edmonson JH, Schomberg PJ (1999). Langerhans cell histiocytosis: diagnosis, natural history, management, and outcome. Cancer.

[6] Minkov M, Grois N, McClain K (2009). Langerhans Cell Histiocytosis. Histiocyte Society Evaluation and Treatment Guidelines.

[7] Kilpatrick SE, Wenger DE, Gilchrist GS (1995). Langerhans’ cell histiocytosis (histiocytosis X) of bone. A clinicopathologic analysis of 263 pediatric and adult cases. Cancer.

[8] Mampaey S, Warson F, Van Hedent E, De Schepper AM (1999). Imaging findings in Langerhans’ cell histiocytosis of the liver and the spleen in an adult. Eur Radiol.

[9] Redhu R, Nadkarni T, Mahesh R (2011). Diabetes insipidus associated with a thickened pituitary stalk in a case of Langerhans cell histiocytosis. J Pediatr Neurosci.

[10] Lieberman PH, Jones CR, Steinman RM (1996). Langerhans cell (eosinophilic) granulomatosis. A clinicopathologic study encompassing 50 years. Am J Surg Pathol.

[11] Broadbent V, Gadner H, Komp DM, Ladisch S (1989). Histiocytosis syndromes in children: II. Approach to the clinical and laboratory evaluation of children with Langerhans cell histiocytosis Clinical Writing Group of the Histiocyte Society. Med Pediatr Oncol.

[12] Minkov M, Grois N, Heitger A (2000). Treatment of multisystem LCH. Results of DAL-HX 83 and DAL-HL 90 studies DAL-HX study group. Klin Padiatr.

